# Mediterranean Diet-Based Sustainable Healthy Diet and Multicomponent Training Combined Intervention Effect on Body Composition, Anthropometry, and Physical Fitness in Healthy Aging

**DOI:** 10.3390/nu16203527

**Published:** 2024-10-18

**Authors:** Joana Sampaio, Andreia Pizarro, Joana Pinto, Bruno Oliveira, André Moreira, Patrícia Padrão, Paula Guedes de Pinho, Pedro Moreira, Renata Barros, Joana Carvalho

**Affiliations:** 1 Faculty of Sport (FADEUP), University of Porto, 4200-450 Porto, Portugal; anpizarro@fade.up.pt; 2 Research Centre in Physical Activity, Health, and Leisure (CIAFEL), University of Porto, 4200-450 Porto, Portugal; pedromoreira@fcna.up.pt; 3 Epidemiology Research Unit (EPIUnit), Public Health Institute (ISPUP), University of Porto, 4050-600 Porto, Portugal; andremoreira@med.up.pt (A.M.); patriciapadrao@fcna.up.pt (P.P.); 4 Laboratory for Integrative and Translational Research in Population Health (ITR), 4050-600 Porto, Portugal; 5 Associate Laboratory Institute for Health and Bioeconomy (i4HB), University of Porto, 4050-313 Porto, Portugal; jipinto@ff.up.pt (J.P.); pguedes@ff.up.pt (P.G.d.P.); 6 Research Unit on Applied Molecular Biosciences (UCIBIO/REQUIMTE), Laboratory of Toxicology, Department of Biological Sciences, Faculty of Pharmacy, University of Porto, 4050-313 Porto, Portugal; 7 Faculty of Nutrition and Food Sciences (FCNAUP), University of Porto, 4150-180 Porto, Portugal; bmpmo@fcna.up.pt; 8 Faculty of Medicine (FMUP), University of Porto, 4200-319 Porto, Portugal

**Keywords:** Mediterranean diet-based sustainable healthy diet, multicomponent training, body composition, anthropometry, physical fitness, aging

## Abstract

Background: Diet and exercise interventions have been associated with improved body composition and physical fitness. However, evidence regarding their combined effects in older adults is scarce. This study aimed to investigate the impact of a combined 12-week Mediterranean diet-based sustainable healthy diet (SHD) and multicomponent training (MT) intervention on body composition, anthropometry, and physical fitness in older adults. Methods: Diet intervention groups received a weekly SHD food supply and four sessions, including a SHD culinary practical workshop. The exercise program included MT 50 min group session, three times a week, on non-consecutive days. Body composition and physical fitness variables were assessed through dual X-ray absorptiometry, anthropometric measurements, and senior fitness tests. Repeated measures ANOVA, with terms for group, time, and interaction, was performed. Results: Our results showed that a combined intervention significantly lowered BMI and total fat. Also, significant differences between assessments in all physical fitness tests, except for aerobic endurance, were observed. Adjusted models show significant differences in BMI (*p* = 0.049) and WHR (*p* = 0.037) between groups and in total fat (*p* = 0.030) for the interaction term. Body strength (*p* < 0.001), balance tests (*p* < 0.001), and aerobic endurance (*p* = 0.005) had significant differences amongst groups. Considering the interaction term, differences were observed for upper body strength (*p* = 0.046) and flexibility tests (*p* = 0.004 sit and reach, *p* = 0.048 back scratch). Conclusions: Our intervention study demonstrates the potential of implementing healthy lifestyle and sustainable models to promote healthy and active aging.

## 1. Introduction

The global population is aging at an exponential rate, accompanied by increased comorbidities associated with aging. This leads to a progressive and irreversible decline in functionality, autonomy, and quality of life [1]. Unhealthy lifestyle factors (e.g., unhealthy dietary patterns or physical inactivity) can increase or worsen the incidence of age-related comorbidities and can be modifiable to potentially improve health outcomes for the global population [2].

Sustainable healthy diets (SHDs) promote a higher intake of plant-based foods and poorer of animal-based foods, and the Mediterranean diet is an excellent example [3]. Recent studies showed that Mediterranean diet-based SHDs are associated with lower weight, body mass index (BMI), waist circumference (WC), waist-to-hip ratio (WHR), trunk fat, total fat [4], and higher lean mass [5]. A recent meta-analysis demonstrated that higher adherence to the Mediterranean diet in older adults is associated with increased cardiorespiratory, musculoskeletal, and overall physical fitness [6].

Multicomponent training (MT) implies a combination of endurance, strength, balance, coordination, and flexibility exercises and, significantly, relies on aerobic metabolism [7]. It seems to have more beneficial effects than isolated training interventions improving fall risk, mobility, balance, functional ability, muscle strength, and body composition [8]. Furthermore, MT programs promote weight loss and reduce body fat percentage [9].

Studies regarding the combined effect of diet and exercise interventions on older adults’ health, particularly with these two approaches, are scarce. Most of them do not have a practical component (are only based on counselling) nor do they have a continuous offer of a food supply and, typically, they are based on an isolated type of training program (e.g., resistance or aerobic training). Recent systematic reviews and meta-analyses with combined diet and exercise interventions in sarcopenic older adults showed that the combination of resistance exercise and daily protein supplementation improved skeletal muscle mass, total lean mass, strength, stability, and quality of life [10] and decreased fat mass [11]. A randomized controlled trial (RCT) in a diabetic population also observed that a combined intervention decreased body weight, fat mass, and visceral adipose tissue, and improved the physical fitness of the individuals [12]. The existing literature on these two interventions is mainly in older individuals with diseases, relying on educational or advisory programs delivered at hospital, at home, or online [13,14]. However, the combined intervention seems to improve several health indicators to a greater extent, and to reduce mortality and promote a wealthy quality of life [13,14,15,16,17]. Combined diet and exercise interventions have been shown also to be effective in increasing walking and daily moderate-to-vigorous physical activity (MVPA) and in reducing fat intake [18]. Greater improvements in the reduction of all-cause mortality, metabolic syndrome, and cardiovascular risk factors seem to be observed when both healthy behaviors are adopted [19,20]. Still, there are gaps regarding the synergistic effect of combined lifestyle intervention programs on specific physical fitness variables and anthropometric variables, especially when combining the Mediterranean diet and MT programs, and whether this can have a greater effect than the isolated components, particularly regarding strength, as well as improving metabolic risk factors and promoting a more autonomous elderly person.

Therefore, the main purpose of this study was to explore the effectiveness of a Mediterranean diet-based SHD and MT interventions on body composition, anthropometry, and physical fitness in community-dwelling older adults. The hypothesis posits that the combined Mediterranean diet-based SHD and MT intervention will result in significant and additional improvements in body composition and physical fitness. Due to its broad applicability, it is pertinent to assess to what extent a combined Mediterranean diet-based SHD and MT can contribute to better health-related outcomes as an alternative lifestyle approach to mitigate biopsychosocial determinants of aging, raising awareness of the importance and feasibility of these combined interventions as effective public health initiatives.

## 2. Materials and Methods

### 2.1. Study Design and Setting

This quasi-experimental study results from the “Multidimensional Health Impact of Multicomponent Exercise and Sustainable Healthy Diet Interventions in the Elderly (MED-E)” project. The project was based in Oporto Metropolitan Area, North Portugal, and data were collected between November 2021 and December 2022. The MED-E project was approved by the Ethical Committee of the Faculty of Food and Nutrition Sciences of the University of Porto (ref. 17/2021/CEFCNAUP/2021, 10 March 2021), the Ethics Committee of the Northern Region Health Administration (report CE/2022/71, 7 July 2022) and by the Data Protection Unit of the University of Porto (ref. P-9/2022, 18 November 2022). Informed consent and an attached file with information regarding confidentiality and data transfer was obtained from interested participants.

The sample size was estimated based on the previous literature and the ANOVA to analyze the differences between and within groups [21]. A sample size calculation was conducted using G*Power 3.1.3 (Universität Düsseldorf, Düsseldorf, Germany) [22] and based on a moderate effect size of 0.40, an alpha risk of 0.05, and a beta risk of 0.20 in a two-sided test. A total sample of at least 100 community-dwelling older adults (25 participants per group), both men and women, accounting for an anticipated dropout rate of 20%, was estimated. Other details regarding the MED-E study protocol [23] and the project impact on biomarkers, metabolome, and nutrients intake [24], can be found elsewhere.

### 2.2. Participants Eligibility Criteria

Community-dwelling older adults (aged 65 years or over), with autonomous mobility and the absence of any neuropsychiatric, musculoskeletal and/or cardiovascular conditions that contraindicate participation in moderate exercise and testing, were eligible to participate in the study. Individuals already engaged in regular MVPA and/or being followed by a nutritionist [23] were excluded.

Individuals who agreed to participate were assigned into one of the four 12-week intervention groups: Mediterranean diet-based SHD, MT, combined Mediterranean diet-based SHD and MT (SHD + MT), or the control group (CG), according to their availability.

### 2.3. Interventions Description

The diet intervention consisted of a Mediterranean diet-based SHD that, despite being healthy and nutritionally adequate, was also affordable, accessible, traditional, culturally acceptable, safe, and with a low footprint impact. Also, seasonal foods and local producers were preferred whenever possible. SHD and SHD + MT participants received, throughout the intervention period, a weekly traditional and local SHD food supply, richer in plant Mediterranean diet-based foods, guaranteed by a national food retailer chain. In accordance with the latest EAT-Lancet recommendations [3] and the Mediterranean diet’s principles, the food supply comprised 125 g of walnuts; 350 g of dry pulses (based on a daily serving of 50 g, varying from chickpeas to different types of beans); one bottle of extra-virgin olive oil (per month); and two 100 g servings of oily fish (sardine fillets and tuna). The intervention included four sessions, with a duration range of 30–60 min. All sessions aimed to improve participants’ nutrition literacy, providing them with skills that enable healthier, traditional, and seasonal food products choices. There was a Mediterranean diet-based SHD culinary workshop, two theoretical sessions (an introductory one explaining the principles of a Mediterranean diet-based SHD and its benefits in several health outcomes and another on supermarket shopping, food storage, and strategies to tackle food waste), and one individual telephone interview.

The MT exercise sessions were on non-consecutive days, three times a week, and lasted 50 min. The sessions comprised three core dimensions: 8–10 min warm-up (including walking, postural, mobility, and stretching exercises), 30–35 min moderate intensity specific training (comprising balance, coordination, strength, and aerobic conditioning), and a 5-min cooldown (including breathing and stretching exercises).

Participants in the CG had a monthly social activity session, 50 min each, involving a maximum of 25 individuals per session. Two sessions included low-intensity physical activity (PA) and the theoretical one included information on nutrition-related topics (e.g., the principles of a healthy diet). Moreover, CG participants were instructed to follow their usual dietary and exercise routines. Retention and motivation were ensured with weekly phone calls.

The intervention lasted approximately 12 weeks. Specialized and well-trained researchers on exercise and nutrition conducted the evaluations. Participants were tested at two timepoints: prior (T1) and 12 weeks after intervention (T2).

### 2.4. Outcomes

#### 2.4.1. Body Composition and Anthropometry Assessment

Total body scans through dual-energy X-ray absorptiometry (DXA) (QDR 4500/A, Hologic Explorer, version 12.4, Bedford, MA, USA) were performed to assess body composition, specifically total lean, fat mass, and percent of body fat. Lean mass was integrated in the analysis as a muscle mass proxy [25].

Height (with a portable stadiometer) and body mass (with a weighting scale) were recorded, and body mass index (BMI) was calculated using the standard formula: mass (kg)/height^2^ (m).

Anthropometric measures were obtained while subjects were dressed in light clothing without shoes and with a spring-loaded measuring tape. Both measurements were taken in centimeters, with the participant in a standing relaxed position with the arms folded across the thorax, according with international standards [26]. Waist circumference (WC) was measured at the narrowest point between the lower costal border and the iliac crest, in the end of a normal expiration [26]. Hip circumference (HC) was collected at the level of the greatest posterior protuberance of the buttocks, with the measurer standing at the side of the subject [26]. The waist-to-hip ratio (WHR) was computed by the following formula: waist circumference/hip circumference.

#### 2.4.2. Physical Fitness Assessment

A senior fitness test (SFT) was used to evaluate physical fitness, comprising six functional tests, as follows [27]:

(i) Chair stand—the participant was instructed to stand (completely) and sit as many times as possible in 30 s, to assess lower body strength;
(ii) Arm curl—the participant flexes the arm in full range of motion, with their palm up, and then returns to a completely extended arm. The maximum number of repetitions in 30 s is monitored to assess upper body strength;
(iii) Six-minute walk—measures the participants’ aerobic endurance, advising them to walk as quickly as possible. The number of laps (and the respective meters) within the six-minute limit is recorded;
(iv) Chair sit and reach—the participant slowly leans forward, with their leg extended, keeping the spine as straight as possible. The aim is to try to touch the toes by sliding the hands, one on top of the other, and the distance achieved is recorded as a measure of lower body flexibility;
(v) Back scratch—a surrogate of upper body flexibility, as the participant must place their dominant hand on top of the same shoulder, with their palm open and fingers extended, trying to reach the middle of the back as much as possible. The hand of the other arm should be placed behind the back, with the elbow close to the waist and the hand upwards, trying to reach as high as possible, in an attempt to touch or overlap the extended middle fingers of both hands;
(vi) Timed up-and-go (TUG)—the participant must get up from a chair and walk to a cone, walk around it, return to the chair and sit down, as quickly as possible, to assess dynamic balance.

The one-leg balance test was applied to assess static balance, in which participants must stand unassisted on one leg for as long as possible, without touching the other leg or the ground. The maximum time achieved (up to 45 s) was recorded in seconds [28].

Lastly, a digital Jamar hand dynamometer (Sammons Preston Inc., Illinois, IL, USA) was used to evaluate handgrip strength [29], with the final value being the best result of three trials.

#### 2.4.3. Sociodemographic and Additional Assessments

A semi-structured questionnaire was applied to gather data on sociodemographic and economic status, clinical history, tobacco use, medication, cognition [30,31], and quality of life [32,33].

Participants provided the highest qualification completed, and education was subsequently classified in three categories according to years of education (≤3, 4–6, and ≥7).

Also, blood samples were collected, and standard biochemical analysis and untargeted nuclear magnetic resonance (NMR)-based metabolomics were performed in certified laboratories. The results of the MED-E intervention’s impact on clinical parameters is presented elsewhere [24].

#### 2.4.4. Dietary Intake Assessment and Mediterranean Diet Adherence

As recommended by the European Food Safety Authority [34] for this population group, two repeated 24 h dietary recalls, on non-consecutive days, were applied to evaluate dietary intake. The nutritional analysis module eAT24 (an electronic instrument that uses the FoodEx2 classification system [35]) estimated energy and nutritional intake.

Participants’ adherence to the Mediterranean diet was scored through the Mediterranean Diet Adherence Screener (MEDAS). Scores greater than 9 (in a range of 14 points) indicate high adherence to the dietary pattern [36].

#### 2.4.5. Physical Activity

Daily PA was assessed through the short version of the International Physical Activity Questionnaire (IPAQ-SV) [37], already validated in Portuguese [38]. IPAQ-SV data were converted as median MET-minutes/week of vigorous physical activity (VPA), moderate physical activity (MPA), and walking, according to the guidelines [39]. Total PA was computed as the sum of these three variables and expressed in total MET-minutes/week.

### 2.5. Statistical Analysis

Descriptive measures were calculated for all variables. Measures of central tendency and dispersion, presented as mean and standard deviation (SD) and frequencies (percentages), were used as appropriate to describe sample characteristics. Data distribution was verified by analyzing symmetry and flatness.

Pearson’s chi-square test, one-way analysis of variance (ANOVA), and the correspondent non-parametric test (Kruskal–Wallis) were used to study significant heterogeneity in the participants baseline characteristics.

Paired samples *t*-Student tests were used to compare means of body composition, anthropometry, and physical fitness tests outcomes at T1 and T2 timepoints in each intervention group. When the variables departed from normality, the related-samples Wilcoxon signed rank test was used instead.

The outcomes were assessed, with normal or log-transformed data (depending on distribution), using repeated measures ANOVA, for differences in main effects (*p*(group) and *p*(time)) and time by group interactions (*p*(time × group)). The association models are presented with crude and adjusted data, considering sex, age, total energy intake, and physical activity variables as covariates. Although education and occupation were initially considered potential covariates, they were ultimately excluded from the final adjusted models since there were no statistically significant baseline differences between groups. Pairwise comparisons used the Sidak post hoc test.

All statistical procedures were executed with Statistical Package for the Social Sciences (SPSS)^®^ software, version 29.0 (IBM, Armonk, NY, USA), considering a significance level of 0.05.

## 3. Results

### 3.1. Participants Characteristics

Participants’ baseline characteristics are presented in Table 1. No significant differences were found at baseline, except for the BMI and usual physical activity, demonstrating a homogeneity amongst participants of the four intervention groups. Most of the participants were female (73.6%), married (58.6%), and retired (97.7%). Participants’ education attainment was lower than six years in 40.2%, with a mean of approximately 9 ± 4 years, and almost all were non-smokers (98.9%). Baseline BMI showed statistically significant differences between groups: SHD + MT participants presented the lowest values. The total mean energy intake was 1726 (±570) kcal/day, with higher intakes observed in the CG participants, but these were not statistically significant. Also, although no significant differences were observed in macronutrients intake (except for saturated fat), added sugars intake was higher in SHD, and total and saturated fat presented higher values in CG. Baseline adherence to the Mediterranean diet was moderate and similar between groups, with a mean score of 9 ± 2 (however, SHD and SHD + MT presented higher scores, representing a good/very good adherence at baseline). There were significant baseline differences in the daily physical activity levels: the SHD + MT had higher levels for habitual moderate-to-vigorous physical activity (MVPA) and walking, especially when compared to CG and SHD.

Of the 87 participants submitted to T1 assessments, 3 participants dropped out from the CG, resulting in incomplete T2 assessments. There were no statistically significant differences between the dropout and intervention groups participants for any descriptive parameters. The reasons for dropping out (illness) were not concomitant to possible adverse effects of the program.

### 3.2. Body Composition and Anthropometry

Table 2 demonstrates that all the intervention groups, especially the MT and SHD + MT groups, exhibited notable improvements in their body composition and anthropometric outcomes. The SHD + MT and MT groups were the ones that improved all the body composition and anthropometric measures (statistically significant differences in BMI (*p* = 0.005 and *p* = 0.002), total fat percent (*p* = 0.007 and *p* = 0.043), and total fat mass (*p* = 0.009 and *p* = 0.033). For the CG and SHD, there were no significant differences between assessment timepoints. However, we can observe an increase in BMI in CG participants. WHR remained similar for CG participants and decreased in the other groups. Total fat mass increased for SHD participants, and lean mass decreased.

Table 3 shows that, when adjusting data for all the crucial covariates (adjusted model 2), BMI changes remained statistically significant only between groups (*p* = 0.049) and total fat (percent and mass) exhibited changes for the interaction term (*p* = 0.030). WHR maintains its significant changes for groups (with SHD participants presenting significantly lower values than CG—Table 4). The significance of lean mass dissipated after adjustment. Table 5 shows that, when considering the interaction term, significant decreases along the intervention are observed in the combined intervention group. Also, total fat percent and mass have significant improvements in the SHD + MT and MT participants (Table 5).

### 3.3. Physical Fitness

Regarding physical fitness outcomes, SHD + MT participants significantly improved their performance in all the physical fitness tests between assessment timepoints, except for aerobic endurance (*p* = 0.426). For the MT participants, significant differences were observed in lower body flexibility (sit and reach: *p* = 0.003), balance tests (*p* = 0.030), and handgrip strength (*p* = 0.026). CG participants were the only ones on the balance tests that did not improve their results, as well as in the lower body flexibility (sit and reach test) in CG and SHD participants. Lower body, upper body, handgrip strength, and aerobic endurance were improved in all groups, although some did not present significant differences.

When adjusting the model for all the covariates (Table 3), significance remained between groups only for upper body and lower body strength and balance (*p* < 0.001): CG and SHD had significantly worse results in the physical fitness tests in comparison to MT and SHD + MT participants (Table 4). Table 3 shows that aerobic endurance (*p* = 0.005) was only significantly different between SHD and MT participants, with the latter group having better results. Static balance (*p* < 0.001) was also significantly lower in SHD when compared to MT and SHD + MT participants—Table 4. Additionally, the interaction term revealed significant changes for aerobic endurance and upper and lower body flexibility (*p* = 0.046, the back scratch test: *p* = 0.048, and the sit and reach test: *p* = 0.004, respectively). Table 5 demonstrates that significant improvements in the physical fitness tests are observed mainly in the SHD + MT and MT groups.

## 4. Discussion

The 12-week combined Mediterranean diet-based sustainable healthy diet and multicomponent training intervention resulted in beneficial and additional improvements in body composition, anthropometry, and physical fitness in community-dwelling older adults.

Significant differences were observed between groups for BMI and WHR and when considering the interaction term for total fat percent and mass. In general, participants from all intervention groups improved their physical fitness results, although some of them did so without statistical significance. Body strength, aerobic endurance, and balance tests showed significant differences between groups and when considered the interaction term differences were observed for the upper body strength and flexibility tests.

Previous findings corroborate our results, showing that combining diet interventions with moderate-to-vigorous physical activity (MVPA), resulted in greater body weight and fat reduction while maintaining or increasing the lean mass [40]. In the present study, we can observe that the SHD + MT and MT participants improved all the body composition and anthropometric parameters. This shows that diet itself does not have such a strong potential to significantly modulate these outcomes [41]. The previous literature demonstrated that combining a greater adherence to the Mediterranean diet with better physical fitness promotes significant improvements in body weight, WC, WHR, and total fat [42]. The higher intake of plant-based foods (e.g., fruit and vegetables) and higher levels of PA seem to be related to a decreased risk of being overweight or obese [43] and, therefore, may contribute to weight loss or maintenance. Although our intervention encouraged a higher intake of plant-based products, pulses and nuts were the promoted foods. Also, the level of the PA sessions was moderate.

Abdominal obesity can usually be evaluated by either WC or WHR [44]. Significant changes were observed between groups in the WHR. However, this parameter decreased (non-statistically significant) after 12 weeks in the MT, SHD, and SHD + MT groups and remained the same in CG: significant differences were only observed between CG and SHD participants. To substantially improve WC, and subsequently impact WHR, a longer intervention period may be necessary. Previous studies with extended intervention periods (6 to 12 months) observed significant circumference reductions [45], as well as shorter-term studies promoting hypocaloric and energy or nutrient restricted diets [11].

A study that combined a high-protein diet with a resistance training program observed that only combined intervention could effectively preserve lean body mass [46]. Nonetheless, no significant differences in total lean mass were observed in our study, which can be attributed to the exercise sessions’ insufficient intensity [40], lower volume of resistance training [47], or the participants’ better baseline body composition. This parameter increased in all groups except for SHD, where it remained approximately the same. As such, it appears that a SHD intervention contributes to maintaining lean mass. Also, the literature states that combined exercise training programs can produce a concurrent training effect, in which aerobic training can delay the development of maximum strength and muscle mass [48]. Furthermore, older adults often experience a blunted muscle production, suggesting a potential need for an overall higher protein intake [49]. Previous studies showed that the combination of exercise with protein or *n*-3 polyunsaturated fatty acids (PUFA) seems to be more effective in the enhancement of total lean mass [50,51,52,53]. Although our intervention mixed food supply promoted intake of oily fish and walnuts and plant-based protein foods, providing protein and different types of *n*-3 PUFA intake, no higher protein intake above the recommendations was suggested, considering that this was not the aim of this study. However, it is noteworthy that all intervention groups presented mean protein intakes within the recommended values of 0.83 g of protein per kg of total body weight per day [24,54]. In the traditional Mediterranean diet, the contribution of protein intake to total energetic value (TEV) is balanced, at approximately 10–12%, with a moderate contribution from plant-based, fish, and poultry sources and a reduced intake of red and processed meat. This dietary pattern has been associated with greater longevity and health of populations [3]. Therefore, the intervention proposed a total protein intake in low to moderate amounts, in accordance with these standards and age group, and considering the main aim of investigating the impact on overall health status in the elderly. However, previous evidence debated higher protein intake for elderly (≥1–1.2 g/kg/day) to sustain muscle mass, strength, and function and to prevent sarcopenia and frailty in older individuals [55,56]. Our participants have a mean age of 72 years old and are physically active. Despite not having statistical significance, lean mass increased in all intervention groups (except for SHD). In the future, it would be interesting to analyze the impact of a similar intervention but with a resistance training program, which has been well described in the literature for its potential to promote an increase in lean mass.

The combined intervention group achieved significantly better physical fitness results (except for handgrip strength), which aligns with previous studies [13,14]. Also, it was observed that participants from groups with the exercise intervention (MT and SHD + MT groups) had significantly better results than the individuals from the CG or SHD group, demonstrating that exercise is a crucial factor in improving physical fitness results, as expected. When comparing data from both exercise groups, we can observe that SHD + MT participants had a higher improvement and better performance, except for lower body flexibility.

To the best of our knowledge, this may be the first study in an older urban South European population that has investigated the effects of a combined Mediterranean diet-based SHD and MT interventions on body composition, anthropometry, and physical fitness. There is a lack of studies in the literature that combine these two intervention approaches, namely a sustainable healthy dietary pattern and, specifically, a multicomponent training program (most studies include isolated types of training, e.g., resistance or aerobic training). Both outcomes were assessed through a combined approach, which includes both subjective—two repeated 24 h recall questionnaires, i.e., the Mediterranean Diet adherence score and IPAQ-SV questionnaire, and objective measurement techniques, i.e., DXA canning. The utilisation of DXA scanning as a gold-standard method further bolsters the robustness of our methodology. Furthermore, it should be highlighted that our Mediterranean diet-based SHD intervention featured a practical component (a Mediterranean diet workshop) alongside the provision of a complete mixed food supply comprising nuts, pulses, extra-virgin olive oil, and oily fish, throughout the 12-week intervention, which can contribute to better nutritional adequacy through the provision and promotion of Mediterranean diet-based healthier food items. The latest nutrition and exercise recommendations for this population group were followed. Also, using the exact same intervention location for all participants can help minimize bias in the study. Moreover, MED-E aimed to investigate the impact of these interventions in a major and broader dimension: clinical, cognitive, and quality of life data were also assessed.

Some limitations of this study must be acknowledged. A crossover and randomized trial design was expected but, due to recruitment and time challenges during and after the long pandemic situation, the study design had to be restructured. As such, the lack of randomization inhibits the possibility of establishing a causal relationship. Because our sample was not representative of Portuguese older population, generalizability of our results is not feasible. Similarly, type I errors due to our multivariate analysis and comparisons can also occur. However, it is noteworthy that the resulting groups did not display significant heterogeneity, which empowers the study. The 24 h recall repeated questionnaires rely on very recent memory, which can bias the data collected regarding dietary intake. Nevertheless, the study followed the international recommendations from the European Food Safety Authority [34] in multicentric studies with adults to assess dietary intake. Participants already had higher levels of MVPA at baseline. However, we accounted for this possible bias by adding these variables as covariates in the association models. Also, an objective assessment of PA was not achievable, as many participants refused to use the accelerometer for the seven days required.

## 5. Conclusions

In summary, the data show that a 12-week combined Mediterranean Diet-based SHD and MT intervention program resulted in beneficial and additional improvements in body composition, anthropometry, and physical fitness in community-dwelling older adults. Although longer-term, larger, and randomized clinical trials are essential for validating these findings, our research contributes to broadening public health aging strategies. Given the paramount importance of primary prevention in promoting healthy dietary patterns, such as the Mediterranean diet and active healthy lifestyles, while decreasing the health burden through the promotion of a better body composition and physical fitness contribute to a globally enhanced health status, our findings help to expand public health aging strategies. The program’s vast applicability and feasibility reinforce its potential impact on promoting healthier and successful aging strategies in the community, enhancing a better global quality of life in this population.

## Figures and Tables

**Table 1:** Baseline sample characteristics.

Characteristics	Total (n = 87)	CG (n = 19)	MT (n = 20)	SHD (n = 26)	SHD + MT (n = 22)	*p*-Value
Age (years), mean (SD) ^+^	72 ± 5	71 ± 4	75 ± 5	72 ± 5	71 ± 5	0.083
Age, range	63–88	65–79	66–82	64–88	63–85	
Sex (female), n (%) ^∀^	64 (73.6)	13 (68.4)	16 (80.0)	16 (61.5)	19 (86.4)	0.215
Civil status, n (%) ^∀^						
Single	5 (5.7)	3 (15.8)	-	-	2 (9.1)	0.169
Married	51 (58.6)	11 (57.9)	13 (65.0)	16 (61.5)	11 (50.0)	
Divorced/separated	10 (11.5)	3 (15.8)	-	3 (11.5)	4 (18.2)	
Widowed	21 (24.1)	2 (10.5)	7 (35.0)	7 (26.9)	5 (22.7)	
Years of formal education, mean (SD) ^#^	9 ± 4	11 ± 5	9 ± 4	8 ± 3	9 ± 5	0.320
Education levels, n (%) ^∀^						
≤3 years	3 (3.4)	1 (5.3)	-	-	2 (9.1)	0.372
4–6 years	32 (36.8)	4 (21.1)	9 (45.0)	11 (42.3)	8 (36.4)	
≥7 years	52 (59.8)	14 (73.7)	11 (55.0)	15 (57.7)	12 (54.5)	
Work condition (retired), n (%) ^∀^	85 (97.7)	18 (94.7)	20 (100.0)	26 (100.0)	21 (95.5)	0.509
Current smoker (no), n (%) ^∀^	86 (98.9)	18 (94.7)	20 (100.0)	26 (100.0)	22 (100.0)	0.305
BMI (kg/m^2^), mean (SD) ^+^	28.8 ± 4.3	28.2 ± 4.0	28.5 ± 4.2	30.9 ± 3.9	27.0 ± 4.1	**0.012**
Total energy intake (kcal/day), mean (SD) ^#^	1726 ± 570	1907 ± 675	1693 ± 552	1715 ± 579	1612 ± 468	0.690
Macronutrients intake (% TEV), mean (SD)						
Protein ^#^	17 ± 4	16 ± 3	18 ± 3	17 ± 5	15 ± 3	0.108
Carbohydrates ^+^	54 ± 8	53 ± 7	53 ± 7	54 ± 9	56 ± 8	0.456
Added sugars ^#^	6 ± 5	6 ± 5	6 ± 5	7 ± 4	5 ± 5	0.334
Lipids ^+^	29 ± 7	31 ± 6	29 ± 6	29 ± 8	28 ± 8	0.756
MUFA ^+^	12 ± 3	12 ± 3	12 ± 4	12 ± 3	12 ± 3	0.977
PUFA ^#^	5 ± 2	5 ± 2	5 ± 2	5 ± 2	6 ± 3	0.675
SFA ^+^	9 ± 3	10 ± 3	9 ± 3	8 ± 3	8 ± 3	0.046
MEDAS score, mean (SD) ^#^	9 ± 2	9 ± 2	9 ± 2	10 ± 2	10 ± 1	0.185
Daily physical activity (MET-min/week), mean (SD) ^#^						
Total physical activity	2322 ± 2753	1227 ± 1151	1957 ± 1115	1577 ± 2120	4479 ± 4080	**<0.001**
Vigorous physical activity (VPA)	945 ± 2148	152 ± 438	926 ± 550	594 ± 2048	2064 ± 3380	**<0.001**
Moderate physical activity (MPA)	756 ± 1187	212 ± 473	769 ± 1115	509 ± 673	1505 ± 1727	**<0.001**
Walking	621 ± 792	864 ± 1136	262 ± 392	473 ± 485	910 ± 867	**0.002**
WHOQOL-OLD score, mean (SD) ^+^	101 ± 11	101 ± 12	103 ± 11	99 ± 12	102 ± 10	0.594
MoCA score, mean (SD) ^+^	20 ± 4	21 ± 4	20 ± 5	21 ± 4	20 ± 4	0.602

^+^ Listed *p* values for one-way ANOVA test, ^#^ Listed *p* values for the independent samples Kruskal–Wallis test, ^∀^ Listed *p* values for χ^2^: Pearson’s chi-square test, bolded *p*-values are significant at the 0.05 level. BMI: body mass index, MEDAS: The Mediterranean Diet Adherence Screener Questionnaire, WHOQOL-OLD: The World Health Organization Quality of Life for Old Module, MoCA: Montreal Cognitive Assessment, % TEV: percentage of total energetic value, MUFA: monounsaturated fatty acids, PUFA: polyunsaturated fatty acids, SFA: saturated fatty acids.

**Table 2:** Pre- (T1) and post-intervention (T2) differences in body composition, anthropometry, and physical fitness outcomes differences.

Outcomes	CG		MT		SHD		SHD + MT	
	Mean Difference (T2 − T1)	*p*-Value	Mean Difference (T2 − T1)	*p*-Value	Mean Difference (T2 − T1)	*p*-Value	Mean Difference (T2 − T1)	*p*-Value
BMI (kg/m^2^) ^∋^	0.05 ±0.37	0.599	−0.49 ±0.58	**0.002**	−0.05 ±0.70	0.726	−0.58 ±0.86	**0.005**
WHR (cm) ^∋^	0.01 ±0.05	0.470	−0.02 ±0.04	0.090	−0.01 ±0.05	0.251	−0.02 ±0.04	0.054
Total fat percent (%) ^∋^	−0.09 ±1.58	0.828	−1.03 ±1.87	**0.043**	0.39 ±1.68	0.254	−1.32 ±1.96	**0.007**
Total fat mass (kg) ^∋^	−0.17 ±0.92	0.477	−0.91 ±1.55	**0.033**	0.33 ±1.48	0.284	−1.04 ±1.59	**0.009**
Total lean mass (kg) ^∋^	0.17 ±1.26	0.605	0.40 ±1.38	0.271	−0.36 ±1.47	0.234	0.22 ±1.34	0.465
Chair stand (nr. repetitions) ^∋^	0.50 ±3.08	0.526	1.32 ±4.00	0.169	0.96 ±3.19	0.137	4.64 ±4.23	**<0.001**
Arm curl (nr. repetitions) ^∋^	−0.06 ±3.30	0.941	1.15 ±4.31	0.247	1.54 ±2.63	**0.006**	4.86 ±3.67	**<0.001**
Aerobic endurance (m) ^ε^	42.88 ±99.61	0.069	11.10 ±55.74	0.257	4.70 ±37.93	0.716	0.18 ±85.13	0.426
Sit and reach (cm) ^ε^	−2.03 ±4.58	0.108	5.70 ±7.50	**0.003**	−1.40 ±6.80	0.241	6.10 ±6.90	**<0.001**
Back scratch (cm) ^∋^	0.93 ±3.63	0.336	1.29 ±3.05	0.082	−0.62 ±4.26	0.468	2.68 ±3.78	**0.005**
Dynamic balance (TUG, s) ^ε^	0.12 ±0.95	0.535	−0.41 ±1.06	**0.030**	−0.17 ±1.24	0.057	−0.35 ±0.29	**<0.001**
Static balance (s) ^ε^	−3.33 ±10.47	0.272	8.40 ±16.58	**0.030**	1.10 ±12.85	0.968	8.21 ±14.18	**0.007**
Handgrip strength (kg) ^ε^	0.67 ±1.64	0.117	0.82 ±2.58	**0.026**	0.63 ±3.40	0.403	2.95 ±2.28	**<0.001**

^∋^ Listed *p* values for the paired samples *t*-Student test, ^ε^ Listed *p* values for the related-samples Wilcoxon signed rank test, Bolded *p*-values are significant at the 0.05 level. T1: first timepoint—baseline, T2: second timepoint—post-intervention, end of the study, BMI: body mass index, WHR: waist-to-hip ratio, TUG: timed up-and-go.

**Table 3:** Impact of the intervention time and group on body composition, anthropometry, and physical fitness outcomes in the four experimental groups, with unadjusted and adjusted models.

Outcomes	Unadjusted Model ^•^			Adjusted Model 1 ^••^			Adjusted Model 2 ^•••^		
	*p*(Time)	*p*(Group)	*p* (Time × Group)	*p*(Time)	*p*(Group)	*p* (Time × Group)	*p*(Time)	*p*(Group)	*p* (Time × Group)
Body composition and anthropometry									
BMI (kg/m^2^)	**0.002**	**0.013**	**0.019**	0.832	**0.019**	**0.039**	0.460	**0.049**	0.285
WHR (cm)	0.089	**0.046**	0.339	0.666	0.064	0.373	0.464	**0.037**	0.708
Total fat percent (%)	**0.014**	0.510	**0.007**	0.714	**0.023**	**0.028**	0.784	0.078	**0.030**
Total fat mass (kg)	**0.007**	0.066	**0.007**	0.746	**0.023**	**0.027**	0.740	0.077	**0.030**
Total lean mass (kg)	0.463	**0.027**	0.208	0.439	0.084	0.191	0.261	0.074	0.134
Physical fitness									
Chair stand (nr. repetitions)	**<0.001**	**<0.001**	**0.010**	0.367	**<0.001**	**0.016**	0.979	**<0.001**	0.304
Arm curl (nr. repetitions)	**<0.001**	**<0.001**	**0.003**	0.189	**<0.001**	**0.005**	0.625	**<0.001**	**0.046**
Aerobic endurance (m)	**0.012**	0.051	0.222	0.082	**<0.001**	0.276	0.106	**0.005**	0.347
Sit and reach (cm)	**0.002**	0.678	**<0.001**	0.986	0.433	**<0.001**	0.899	0.429	**0.004**
Back scratch (cm)	**0.020**	0.580	0.058	0.845	0.850	**0.045**	0.893	0.908	**0.048**
Dynamic balance (TUG, s)	**<0.001**	**<0.001**	0.075	0.141	**<0.001**	**0.046**	0.950	**<0.001**	0.099
Static balance (s)	**0.030**	**<0.001**	0.062	0.516	**<0.001**	0.184	0.840	**<0.001**	0.312
Handgrip strength (kg)	**<0.001**	**0.011**	**0.004**	0.741	0.082	**0.007**	0.377	0.091	0.150

^•^ Listed *p* values for within- and between-subject effects assessed by repeated measures ANOVA, unadjusted model, ^••^ Listed *p* values for within- and between-subject effects assessed by repeated measures ANOVA, unadjusted model further adjusted for baseline age (years) and sex, ^•••^ Listed *p* values for within- and between-subject effects assessed by repeated measures ANOVA, adjusted model 1 further adjusted for baseline energy intake (kcal/day) and total physical activity (MET-min/week); bolded *p*-values are significant at the 0.05 level. BMI: body mass index, WHR: waist-to-hip ratio, TUG: timed up-and-go.

**Table 4:** Significant differences between groups through post hoc Sidak pairwise comparisons, based on estimated marginal means for the adjusted model 2.

Outcomes	(I) Participants’ Group Identification	(J) Participants’ Control or Intervention Group Identification	Mean Difference (I − J) *	Std. Error	Sig. ^b^	95% Confidence Interval for Difference ^b^	
						Lower Bound	Upper Bound
WHR (cm)	CG	SHD	−0.05	0.02	0.039	−0.11	−0.00
Chair stand (nr. repetitions)	CG	MT	−6.12	1.22	<0.001	−9.42	−2.82
		SHD + MT	−4.97	1.23	<0.001	−8.30	−1.64
	SHD	MT	−6.87	1.07	<0.001	−9.78	−3.97
		SHD + MT	−5.72	1.07	<0.001	−8.62	−2.82
Arm curl (nr. repetitions)	CG	MT	−4.82	1.38	0.005	−8.56	−1.07
		SHD + MT	−4.35	1.39	0.016	−8.12	−0.57
	SHD	MT	−5.21	1.21	<0.001	−8.51	−1.92
		SHD + MT	−4.74	1.21	0.001	−8.03	−1.44
Aerobic endurance (m)	SHD	MT	−35.15	9.82	0.004	−61.81	−8.48
Dynamic balance (TUG, s)	CG	MT	0.50	0.13	0.002	0.15	0.85
		SHD + MT	0.48	0.13	0.003	0.12	0.83
	SHD	MT	0.56	0.11	<0.001	0.25	0.87
		SHD + MT	0.54	0.11	<0.001	0.22	0.85
Static balance (s)	SHD	MT	−7.75	1.72	<0.001	−12.42	−3.07
		SHD + MT	−5.96	1.72	0.006	−10.64	−1.29

* The mean difference is significant at the 0.05 level, ^b^ Adjustment for multiple comparisons: Sidak. WHR: waist-to-hip ratio, TUG: timed up-and-go.

**Table 5:** Significant differences for the interaction term through post hoc Sidak pairwise comparisons, based on estimated marginal means for the adjusted model 2.

Measure	Participants’ Control or Intervention Group Identification	Mean Difference (T2 − T1) *	Std. Error	Sig. ^b^	95% Confidence Interval for Difference ^b^	
					Lower Bound	Upper Bound
BMI (kg/m^2^)	SHD + MT	−0.53	0.19	0.006	−0.90	−0.15
Total fat percent (%)	MT	−1.16	0.50	0.024	−2.17	−0.16
	SHD + MT	−1.45	0.487	0.004	−2.42	−0.47
Total fat mass (kg)	MT	−0.96	0.41	0.021	−1.78	−0.15
	SHD + MT	−1.18	0.39	0.004	−1.97	−0.40
Chair stand (nr. repetitions)	SHD + MT	3.49	1.02	0.001	1.45	5.53
Arm curl (nr. repetitions)	SHD	1.79	0.81	0.030	0.18	3.40
	SHD + MT	4.42	1.01	<0.001	2.41	6.43
Aerobic endurance (m)	CG	14.18	6.93	0.045	0.33	28.03
Sit and reach (cm)	MT	4.76	1.48	0.002	1.81	7.71
	SHD + MT	5.22	1.43	<0.001	2.37	8.07
Back scratch (cm)	SHD + MT	3.19	1.03	0.003	1.14	5.25
Dynamic balance (TUG, s)	MT	−0.28	0.08	0.001	−0.44	−0.11
	SHD	−0.18	0.06	0.006	−0.31	−0.05
Static balance (s)	MT	2.82	1.14	0.016	0.53	5.11
Handgrip strength (kg)	SHD + MT	1.04	0.25	<0.001	0.55	1.53

* The mean difference is significant at the 0.05 level, ^b^ Adjustment for multiple comparisons: Sidak. BMI: body mass index, TUG: timed up-and-go.

## Data Availability

The original contributions presented in the study are included in the article, and further inquiries can be directed to the corresponding author.

## References

[1] Dogra S., Dunstan D.W., Sugiyama T., Stathi A., Gardiner P.A., Owen N. (2022). Active aging and public health: Evidence, implications, and opportunities. Annu. Rev. Public Health.

[2] Prince M.J., Wu F., Guo Y., Gutierrez Robledo L.M., O’Donnell M., Sullivan R., Yusuf S. (2015). The burden of disease in older people and implications for health policy and practice. Lancet.

[3] Willett W., Rockstrom J., Loken B., Springmann M., Lang T., Vermeulen S., Garnett T., Tilman D., DeClerck F., Wood A. (2019). Food in the Anthropocene: The EAT-Lancet Commission on healthy diets from sustainable food systems. Lancet.

[4] Livingstone K.M., Milte C., Bowe S.J., Duckham R.L., Ward J., Keske M.A., McEvoy M., Brayner B., Abbott G. (2022). Associations between three diet quality indices, genetic risk and body composition: A prospective cohort study. Clin.Nutr..

[5] Di Rosa C., Lattanzi G., Spiezia C., Imperia E., Piccirilli S., Beato I., Gaspa G., Micheli V., De Joannon F., Vallecorsa N. (2022). Mediterranean Diet versus Very Low-Calorie Ketogenic Diet: Effects of Reaching 5% Body Weight Loss on Body Composition in Subjects with Overweight and with Obesity—A Cohort Study. Int. J. Environ. Res. Public Health.

[6] Bizzozero-Peroni B., Brazo-Sayavera J., Martínez-Vizcaíno V., Fernández-Rodríguez R., López-Gil J.F., Díaz-Goñi V., Cavero-Redondo I., Mesas A.E. (2022). High Adherence to the Mediterranean Diet is Associated with Higher Physical Fitness in Adults: A Systematic Review and Meta-Analysis. Adv.Nutr..

[7] Marques E., Carvalho J., Soares J.M., Marques F., Mota J. (2009). Effects of resistance and multicomponent exercise on lipid profiles of older women. Maturitas.

[8] De Labra C., Guimaraes-Pinheiro C., Maseda A., Lorenzo T., Millán-Calenti J.C. (2015). Effects of physical exercise interventions in frail older adults: A systematic review of randomized controlled trials. BMC Geriatr..

[9] Kemmler W., Von Stengel S., Engelke K., Häberle L., Mayhew J.L., Kalender W.A. (2010). Exercise, Body Composition, and Functional Ability A Randomized Controlled Trial. Am. J. Prev.Med..

[10] Hernández-Lepe M.A., Miranda-Gil M.I., Valbuena-Gregorio E., Olivas-Aguirre F.J. (2023). Exercise Programs Combined with Diet Supplementation Improve Body Composition and Physical Function in Older Adults with Sarcopenia: A Systematic Review. Nutrients.

[11] Eglseer D., Traxler M., Embacher S., Reiter L., Schoufour J.D., Weijs P.J.M., Voortman T., Boirie Y., Cruz-Jentoft A., Bauer S. (2023). Nutrition and Exercise Interventions to Improve Body Composition for Persons with Overweight or Obesity Near Retirement Age: A Systematic Review and Network Meta-Analysis of Randomized Controlled Trials. Adv.Nutr..

[12] Celli A., Barnouin Y., Jiang B., Blevins D., Colleluori G., Mediwala S., Armamento-Villareal R., Qualls C., Villareal D.T. (2022). Lifestyle intervention strategy to treat diabetes in older adults: A randomized controlled trial. Diabetes Care.

[13] de Souto Barreto P., Pothier K., Soriano G., Lussier M., Bherer L., Guyonnet S., Piau A., Ousset P.J., Vellas B. (2021). A web-based multidomain lifestyle intervention for older adults: The eMIND randomized controlled trial. J. Prev. Alzheimer’sDis..

[14] Malakou E., Linardakis M., Armstrong M.E.G., Zannidi D., Foster C., Johnson L., Papadaki A. (2018). Combined Effect of Promoting the Mediterranean Diet and Physical Activity on Metabolic Risk Factors in Adults: A Systematic Review and Meta-Analysis of Randomised Controlled Trials. Nutrients.

[15] Cobos-Palacios L., Ruiz-Moreno M.I., Vilches-Perez A., Vargas-Candela A., Muñoz-Úbeda M., Benítez Porres J., Navarro-Sanz A., Lopez-Carmona M.D., Sanz-Canovas J., Perez-Belmonte L.M. (2022). Metabolically healthy obesity: Inflammatory biomarkers and adipokines in elderly population. PLoS ONE.

[16] Hsieh T.-J., Su S.-C., Chen C.-W., Kang Y.-W., Hu M.-H., Hsu L.-L., Wu S.-Y., Chen L., Chang H.-Y., Chuang S.-Y. (2019). Individualized home-based exercise and nutrition interventions improve frailty in older adults: A randomized controlled trial. Int. J. Behav. Nutr. Phys.Act..

[17] Vieira E.R., Cavalcanti F.A.d.C., Civitella F., Hollifield M., Caceres S., Carreno J., Gaillard T., Huffman F.G., Mora J.C., Queiroga M.R. (2021). Effects of exercise and diet on body composition and physical function in older Hispanics with type 2 diabetes. Int. J. Environ. Res. Public Health.

[18] Burke L., Lee A.H., Jancey J., Xiang L., Kerr D.A., Howat P.A., Hills A.P., Anderson A.S. (2013). Physical activity and nutrition behavioural outcomes of a home-based intervention program for seniors: A randomized controlled trial. Int. J. Behav. Nutr. Phys.Act..

[19] Caprara G. (2021). Mediterranean-type dietary pattern and physical activity: The winning combination to counteract the rising burden of non-communicable diseases (NCDS). Nutrients.

[20] Kazemi A., Sasani N., Mokhtari Z., Keshtkar A., Babajafari S., Poustchi H., Hashemian M., Malekzadeh R. (2022). Comparing the risk of cardiovascular diseases and all-cause mortality in four lifestyles with a combination of high/low physical activity and healthy/unhealthy diet: A prospective cohort study. Int. J. Behav. Nutr. Phys.Act..

[21] Borm G.F., Fransen J., Lemmens W.A.J.G. (2007). A simple sample size formula for analysis of covariance in randomized clinical trials. J. Clin.Epidemiol..

[22] Kwon S., Perera S., Pahor M., Katula J.A., King A.C., Groessl E.J., Studenski S.A. (2009). What is a meaningful change in physical performance? Findings from a clinical trial in older adults (The LIFE-P study). J.Nutr. Health Aging.

[23] Sampaio J., Carvalho J., Pizarro A., Pinto J., Moreira A., Padrão P., Guedes de Pinho P., Moreira P., Barros R. (2023). Multidimensional Health Impact of Multicomponent Exercise and Sustainable Healthy Diet Interventions in the Elderly (MED-E): Study Protocol. Nutrients.

[24] Sampaio J., Pinto J., Pizarro A., Oliveira B., Moreira A., Padrao P., Moreira P., Guedes de Pinho P., Carvalho J., Barros R. (2024). Combined mediterranean diet-based sustainable healthy diet and multicomponent training intervention impact on plasma biomarkers and metabolome in older adults. Clin. Nutr..

[25] Cheung C.-L., Lee G.K.-Y., Au P.C.-M., Li G.H.-Y., Chan M., Li H.-L., Cheung B.M.-Y., Wong I.C.-K., Lee V.H.-F., Mok J. (2021). Systematic review and meta-analysis of lean mass and mortality: Rationale and study description. Osteoporos. Sarcopenia.

[26] Norton K.I. (2018). Standards for anthropometry assessment. Kinanthropometry and Exercise Physiology.

[27] Rikli R.E., Jones C.J. (2001). Senior Fitness Test Manual.

[28] Fregly A.R., Graybiel A. (1968). An ataxia test battery not requiring rails. Aerosp.Med..

[29] Mathiowetz V., Weber K., Volland G., Kashman N. (1984). Reliability and validity of grip and pinch strength evaluations. J. HandSurg..

[30] Duro D., Simoes M.R., Ponciano E., Santana I. (2010). Validation studies of the Portuguese experimental version of the Montreal Cognitive Assessment (MoCA): Confirmatory factor analysis. J. Neurol..

[31] Nasreddine Z.S., Phillips N.A., Bedirian V., Charbonneau S., Whitehead V., Collin I., Cummings J.L., Chertkow H. (2005). The Montreal Cognitive Assessment, MoCA: A brief screening tool for mild cognitive impairment. J. Am. Geriatr. Soc..

[32] Vilar M., Sousa L.B., Simoes M.R. (2016). The European Portuguese WHOQOL-OLD module and the new facet Family/Family life: Reliability and validity studies. Qual Life Res..

[33] World Health Organization (2006). The WHOQOL-OLD module—Manual. https://www.ufrgs.br/qualidep/images/Whoqol-OLD/WHOQOL-OLD_manual_Ingles.pdf.

[34] (2009). European Food Safety Authority, General principles for the collection of national food consumption data in the view of a pan-European dietary survey. EFSAJ..

[35] Lopes C., Torres D., Oliveira A., Severo M., Guiomar S., Alarcao V., Ramos E., Rodrigues S., Vilela S., Oliveira L. (2018). National Food, Nutrition, and Physical Activity Survey of the Portuguese General Population (2015–2016): Protocol for Design and Development. JMIR Res. Protoc..

[36] Gregório M.J., Rodrigues A.M., Salvador C., Dias S.S., De Sousa R.D., Mendes J.M., Coelho P.S., Branco J.C., Lopes C., Martínez-González M.A. (2020). Validation of the Telephone-Administered Version of the Mediterranean Diet Adherence Screener (MEDAS) Questionnaire The telephone-administered version of MEDAS is a valid tool for assessing the adherence to the Mediterranean diet and acquiring data for large population-based studies. Nutrients.

[37] Lee P.H., Macfarlane D.J., Lam T.H., Stewart S.M. (2011). Validity of the International Physical Activity Questionnaire Short Form (IPAQ-SF): A systematic review. Int. J. Behav. Nutr. Phys. Act.

[38] Craig C.L., Marshall A.L., Sjöström M., Bauman A.E., Booth M.L., Ainsworth B.E., Pratt M., Ekelund U., Yngve A., Sallis J.F. (2003). International physical activity questionnaire: 12-Country reliability and validity. Med. Sci. SportsExerc..

[39] IPAQ Research Committee (2005). Guidelines for Data Processing and Analysis of the International Physical Activity Questionnaire (IPAQ)-Short and Long Forms.

[40] Hernández-Reyes A., Cámara-Martos F., Molina-Luque R., Romero-Saldaña M., Molina-Recio G., and Moreno-Rojas R. (2019). Changes in body composition with a hypocaloric diet combined with sedentary, moderate and high-intense physical activity: A randomized controlled trial. BMC Women’s Health.

[41] Chen C.-N., Hsu K.-J., Chien K.-Y., Chen J.-J. (2021). Effects of Combined High-Protein Diet and Exercise Intervention on Cardiometabolic Health in Middle-Aged Obese Adults: A Randomized Controlled Trial. Front. Cardiovasc.Med..

[42] Marcos-Pardo P.J., González-Gálvez N., Espeso-García A., Abelleira-Lamela T., López-Vivancos A., Vaquero-Cristóbal R. (2020). Association among Adherence to the Mediterranean Diet, Cardiorespiratory Fitness, Cardiovascular, Obesity, and Anthropometric Variables of Overweight and Obese Middle-Aged and Older Adults. Nutrients.

[43] Dominguez L.J., Veronese N., Di Bella G., Cusumano C., Parisi A., Tagliaferri F., Ciriminna S., Barbagallo M. (2023). Mediterranean diet in the management and prevention of obesity. Exp.Gerontol..

[44] Qian X., Su C., Zhang B., Qin G., Wang H., Wu Z. (2019). Changes in distributions of waist circumference, waist-to-hip ratio and waist-to-height ratio over an 18-year period among Chinese adults: A longitudinal study using quantile regression. BMC Public Health.

[45] Di Renzo L., Cinelli G., Dri M., Gualtieri P., Attinà A., Leggeri C., Cenname G., Esposito E., Pujia A., Chiricolo G. (2020). Mediterranean personalized diet combined with physical activity therapy for the prevention of cardiovascular diseases in Italian women. Nutrients.

[46] Lockard B., Mardock M., Oliver J.M., Byrd M., Simbo S., Jagim A.R., Kresta J., Baetge C.C., Jung Y.P., Koozehchian M.S. (2022). Comparison of Two Diet and Exercise Approaches on Weight Loss and Health Outcomes in Obese Women. Int. J. Environ. Res. Public Health.

[47] Thomas M.H., Burns S.P. (2016). Increasing Lean Mass and Strength: A Comparison of High Frequency Strength Training to Lower Frequency Strength Training. Int. J. Exerc.Sci..

[48] Lundberg T.R., Feuerbacher J.F., Sünkeler M., Schumann M. (2022). The Effects of Concurrent Aerobic and Strength Training on Muscle Fiber Hypertrophy: A Systematic Review and Meta-Analysis. Sports Med..

[49] Voulgaridou G., Papadopoulou S.D., Spanoudaki M., Kondyli F.S., Alexandropoulou I., Michailidou S., Zarogoulidis P., Matthaios D., Giannakidis D., Romanidou M. (2023). Increasing Muscle Mass in Elders through Diet and Exercise: A Literature Review of Recent RCTs. Foods.

[50] Brook M.S., Din U., Tarum J., Selby A., Quinlan J., Bass J.J., Gharahdaghi N., Boereboom C., Abdulla H., Franchi M.V. (2021). Omega-3 supplementation during unilateral resistance exercise training in older women: A within subject and double-blind placebo-controlled trial. Clin. Nutr. ESPEN.

[51] Mertz K.H., Reitelseder S., Bechshoeft R., Bulow J., Højfeldt G., Jensen M., Schacht S.R., Lind M.V., Rasmussen M.A., Mikkelsen U.R. (2021). The effect of daily protein supplementation, with or without resistance training for 1 year, on muscle size, strength, and function in healthy older adults: A randomized controlled trial. Am. J. Clin.Nutr..

[52] Seino S., Sumi K., Narita M., Yokoyama Y., Ashida K., Kitamura A., Shinkai S. (2018). Effects of Low-Dose Dairy Protein Plus Micronutrient Supplementation during Resistance Exercise on Muscle Mass and Physical Performance in Older Adults: A Randomized, Controlled Trial. J.Nutr. Health Aging.

[53] ten Haaf D.S.M., Eijsvogels T.M.H., Bongers C.C.W.G., Horstman A.M.H., Timmers S., de Groot L.C.P.G.M., Hopman M.T.E. (2019). Protein supplementation improves lean body mass in physically active older adults: A randomized placebo-controlled trial. J. Cachexia Sarcopenia Muscle.

[54] European Food Safety Authority Panel on Dietetic Products (NDA) (2012). Scientific Opinion on Dietary Reference Values for protein. EFSAJ..

[55] Deutz N.E., Bauer J.M., Barazzoni R., Biolo G., Boirie Y., Bosy-Westphal A., Cederholm T., Cruz-Jentoft A., Krznaric Z., Nair K.S. (2014). Protein intake and exercise for optimal muscle function with aging: Recommendations from the ESPEN Expert Group. Clin. Nutr..

[56] Traylor D.A., Gorissen S.H.M., Phillips S.M. (2018). Perspective: Protein Requirements and Optimal Intakes in Aging: Are We Ready to Recommend More Than the Recommended Daily Allowance?. Adv. Nutr..

